# Recent progress in patent foramen ovale and related neurological diseases: A narrative review

**DOI:** 10.3389/fneur.2023.1129062

**Published:** 2023-03-27

**Authors:** Fanfan Shi, Leihao Sha, Hua Li, Yusha Tang, Litao Huang, Huizhen Liu, Xu Li, Lin Li, Wenjie Yang, Deying Kang, Lei Chen

**Affiliations:** ^1^Department of Clinical Research Management, West China Hospital, Sichuan University, Chengdu, China; ^2^Department of Neurology and Joint Research Institution of Altitude Health, West China Hospital, Sichuan University, Chengdu, China; ^3^Department of Evidence-Based Medicine and Clinical Epidemiology, West China Hospital, Sichuan University, Chengdu, China

**Keywords:** patent foramen ovale, stroke, migraine, obstructive sleep apnea, dementia, decompression sickness

## Abstract

Patent foramen ovale (PFO) is a common congenital cardiac abnormality when the opening of the interatrial septum is not closed in adulthood. This abnormality affects 25% of the general population. With the development of precision medicine, an increasing number of clinical studies have reported that PFO is closely related to various neurological diseases such as stroke, migraine, obstructive sleep apnea, and decompression syndrome. It has also been suggested that PFO closure could be effective for preventing and treating these neurological diseases. Therefore, increasing attention has been given to the prevention, diagnosis, and treatment of PFO-related neurological diseases. By reviewing existing literature, this article focuses on the pathogenesis, epidemiology, and clinical characteristics of PFO-related neurological diseases, as well as the prevention and treatment of different neurological diseases to discuss, and aims to provide current progress for this field and decision-making evidence for clinical practice.

## Introduction

The foramen ovale is an opening in the interatrial septum in the fetal heart. After the fetus is born, with the establishment of pulmonary circulation, the left atrial pressure increases, pushing the primary septum to fuse with the secondary septum and then prompting the foramen ovale to close. Foramen ovale closes before the age of 2 years in most people, and approximately 25% fail to close in adulthood, resulting in the formation of congenital heart abnormalities—patent foramen ovale (PFO) (1).

An increasing number of studies have reported that PFO is closely related to many neurological diseases, such as stroke, migraine, obstructive sleep apnea (OSA), and decompression sickness (DCS) (2). For stroke, large-scale clinical studies have suggested that PFO could be a novel risk factor for cryptogenic stroke, especially for younger adults. Three randomized controlled trials (RCTs) from the New England Journal of Medicine also indicated that PFO closure was effective in preventing stroke recurrence (3–5). Based on these, the concept of “PFO-related stroke” was separately proposed in the U.S. SCAI (Society for Cardiovascular Angiography and Interventions) guidelines in 2022, strongly recommending that PFO closure should be used for patients aged 18–60 years with previous PFO-related stroke to prevent stroke recurrence (6). However, some studies have shown that PFO closure has poor therapeutic effects in patients with long-tunnel PFO (7), and the best beneficiaries are not yet clear. For migraine, several observational clinical studies have shown an association with PFO, especially for migraine with aura. However, the efficacy of PFO closure was mixed in three RCTs (8–10), and *post-hoc* analysis found PFO closure was only effective in migraine with aura. The relationship between PFO and migraine remains questionable. For other neurological diseases (such as OSA and DCS), even though some previous literature has also explored the association with PFO, limited evidence supported it. Since neurological diseases are a major burden to human health, and PFO could be a potential novel risk factor and treatment target for neurological diseases, it is very necessary to focus on and carry out related research.

Based on the earlier mentioned text, this article reviews the research progress of PFO diagnosis, as well as epidemiology, clinical characteristics, pathogenesis, prevention, and treatment of PFO-related neurological diseases to provide strong supporting evidence to carry out future research in this field.

## The diagnosis of PFO

Patent foramen ovale was first discovered by autopsy in 1877 (11), and the prevalence reported ranged from 15–35% (12–14). In the 1980s, with the advances of echocardiography and aerated saline contrast media, screening for PFO became common in clinical practice. Common diagnostic techniques include transesophageal echocardiography contrast-enhanced acoustics (cTEE), transthoracic echocardiography contrast-enhanced acoustics (cTTE), and transcranial Doppler contrast-enhanced ultrasound (cTCD), each of which has advantages and disadvantages (refer to details in Table 1). cTEE is the best tool for obtaining an anatomical confirmation of PFO and provides some essential details such as measure (width and length) and presence of an atrial septal aneurysm, and these characteristics have important guidance for treatment decision-making. However, cTEE is also limited by high cost and poor patient tolerance. Therefore, it is not suitable for primary screening. cTTE and cTCD are often presented as potential screening tools. But according to the results of a meta-analysis of diagnostic tests, compared with cTEE, the pooled sensitivity and specificity for cTTE were just 45.1% and 99.6%, and the respective measures for cTCD were 96.1% and 92.4% (18). In view of the lower sensitivity of cTTE, and cTCD can only detect right to left shunt (RLS), but cannot confirm the presence of PFO, the current clinical guidelines of several countries suggest the combination of multiple diagnostic screening tools for screening and diagnosis of PFO (15, 19). Generally, when using cTTE or cTCD, it is recommended to detect both resting and implementing Valsalva. If either item has many microbubbles, there is no need to repeat the examination. If it is negative and PFO is still suspected, it is recommended to (I) repeat Valsalva or cough at an appropriate time (three–four times) for contrast media injection detection of normal saline, (II) use a mixture of blood saline and air, and (III) use TEE detection (image acquisition should begin before the appearance of saline contrast medium in the right atrium and continue for at least 10 cardiac cycles after the appearance of contrast medium) (20).

**Table 1:** Comparison of different diagnostic techniques for PFO.

**Diagnosis method**	**Application**	**Diagnostic criteria (15)**	**Sensitivity/ Specificity**	**Advantages**	**Disadvantages**	**General operation process**
Transthoracic Echocardiography (TTE)	• Observe morphological characteristics of atrial septum and atrial structures •Evaluate potential causes of cardiogenic embolism • Diagnosis of clinically relevant intracardiac shunt	• < 20 bubbles: mild/moderate • >20 bubbles: severe	• 46–100%/ • 85–100%	• Strong operability (16) • Low expense • Repeatable • Visualization and semi-quantization of right-to-left shunt	• Low sensitivity for small RLS shunt • Need an adequate thoracic ultrasound window	• **Preparation of contrast media** Mix 8 ml of normal saline with 1ml blood from the patients and 1 ml of room air in a syringe. • **Injected contrast** Patient is decubitus on the left side, and agitated saline is injected vertically into the left cubit vein at rest and Valsalva maneuver (VM). • **Evaluating Valid VM** (1) cTTE is assessed by a decrease in flow velocity of at least 20 cm/SEC during early transmission (E). (2) cTEE is assessed by looking at the convexity of the left atrial septum and the size of the gap between the original and posterior septum of the atrial septum. (3) cTCD is assessed by monitoring a >25% decrease in peak Doppler flow velocity in the middle cerebral artery. • **Diagnose** (1) cTTE or cTEE confirmed the diagnosis when left ventricular micro-bubbles appeared within first 3–5 cardiac cycles after right ventricular opacification. (2) cTCD confirmed the diagnosis when at least 1 micro bubble appears < 25 s after injection with stirred saline.
Transesophageal Echocardiography (TEE)	• Observe morphological characteristics of atrial septum and atrial structures • Observe PFO anatomical characteristics and to guide PFO closure	• < 20 bubbles: mild/moderate • >20 bubbles: severe	• 67–100%/ • 90–100%	• Gold standard • Semi-quantitative of right-to-left shunt • Distinguish PFO, atrial septal defect, pulmonary artery fistula • Evaluate the size of PFO (17)	• Semi-invasive and uncomfortable • Valsalva operation is limited • More expensive • Easy to underestimate the severity of RLS shunting	
Transcranial Doppler (TCD)	• Diagnosis right-to-left shunt after rest and Valsalva	• 3–10 HITS:mild/moderate • >10HITS: severe	• 68–100%/ • 65–100%	• Good patient cooperation • Low expense • Repeatable • The sensitivity to RLS is the highest • Semi-quantitative of the degree of shunt	• Difficult to distinguish PFO, atrial septal defect, pulmonary arteriovenous	

In terms of any diagnostic techniques, it is essential to strictly follow the operation process. Clinical practice and research have found that the accuracy of PFO diagnosis is easily affected by the operational details, and even the gold-standard cTEE may have a false negative rate of 7.9% due to inadequate pressures generated during Valsalva maneuvers (21). In addition, although some countries have issued corresponding guidelines or consensus (22, 23), there are still disputes over some details. For example, the choice of Valsalva implementation time during cTCD, although most recommended after contrast agent (CA) injection, some studies recommended before or during CA injection and different implementation times, will lead to completely different examination results (24, 25). Another example, most previous studies diagnosed PFO by echocardiography only using left heart contrast and cardiac cycles after right atrium (RA) opacification, but the definition for quantity of left heart contrast and cardiac cycles varies among studies (20). All these problems need more detailed and standardized clinical practice guidelines to guide them.

It is worth mentioning that with the innovation of diagnostic technology, intracardiac echocardiography (ICE) has been gradually explored in the diagnosis and PFO closure. Jeonggeun Moon et al. compared the procedural efficacy and safety of TEE-guide and ICE-guide PFO device closure for the first time and found that the fluoroscopy time, radiation dose, and total procedural time in the catheter laboratory were significantly lower in the ICE group than those in the TEE group while achieving similar procedural outcomes and hospital stay duration (26). Compared with TEE and TTE, ICE has a higher image resolution and can accurately assess the size, location, and edge of PFO from different angles, which makes it easy to capture anatomical information such as atrial myxoma, Chiari network with thrombi, and additional septal defects (27). The development of this technology will bring new prospects for PFO diagnosis and closure. However, the possible disagreement in the anatomical evaluation of PFO between preprocedural TEE and intraprocedural ICE needs to be further studied and considered.

## Epidemiological and clinical characteristics of PFO-related neurological diseases

Thus far, stroke and migraine are the most discussed diseases in the research of PFO-related neurological diseases. According to the results from community-based and multi-center epidemiological studies conducted in different countries, the prevalence of PFO or RLS for stroke ranges from 23.5 to 61.1% (28–32), for migraine with aura from 19 to 77.9% (33–36), and for migraine without aura from a relatively low 11–34.1% (33, 34). The prevalence of patients in most studies was generally greater than the general population. Furthermore, the results of two meta-analyses showed that compared with healthy people, the prevalence of PFO was higher in patients with stroke and migraine (OR = 3.1 and 2.54, respectively) (37, 38), which indicates that there is a close relationship. Studies on the correlation between PFO and OSA, DCS, and dementia (refer to Supplementary Table 1) are relatively few. Some retrospective studies showed the prevalence of PFO in OSA was higher than that in healthy controls (39, 40), and the incidence of PFO in DCS patients is 62.5% (41). In addition, more direct evidence found that venous to arterial circulation shunt (v-aCS) of PFO was more common in both patients with AD and vascular dementia (VaD) than in healthy controls, suggesting that PFO is associated with cognitive dysfunction, especially AD (42, 43). These are worthy of further exploration.

Patent foramen ovale-related strokes were mainly cryptogenic stroke (CS) or embolic stroke of unknown origin (ESUS). According to the proportion of CS in ischemic stroke (44) and the global incidence of ischemic stroke in 2019 (45), the incidence of PFO-related stroke is estimated to be 19 to 28 cases per 100 000 and is more common in people under 55 years of age (46). For elderly patients, Mazzucc. et al. found the prevalence of RLS with cryptogenic transient ischemic attacks and non-disabling strokes reached 28.21% (31). It also needs attention. At present, there is no sex or racial difference in the prevalence of PFO-related stroke, but the incidence of stroke in young women is increasing, which may be caused by risk factors such as blood coagulation and hormonal changes during pregnancy (47). For pregnant women with PFO, the risk of recurrent stroke is higher (48), which deserves more attention too. Migraine is the second leading cause of non-fatal burden globally (49), and few studies reported the unique epidemiological characteristics of PFO-related migraine. But unlike most previous studies, two cross-sectional studies conducted in China found that the incidence of migraine without aura in PFO was significantly higher than in healthy people, suggesting that PFO may be also associated with migraine without aura (50, 51). The correlation deserves further exploration in other countries and populations.

The results of some clinical studies indicated that clinical characteristics and symptoms of PFO-related neurological diseases might be related to the volume of RLS shunt. PFO-related stroke lesions mostly involve vertebrobasilar circulation (52). The greater the amount of RLS shunts, the higher the proportion of small lesions, the greater the likelihood of posterior circulation involvement, and the greater the frequency of multiple cortical lesions (53). But for migraines, the findings were inconsistent. Some studies have shown that the frequency, intensity, and duration of headaches in migraine patients with moderate and severe RLS shunting were significantly higher than those in patients with mild RLS and patients with non-RLS (54), but some found the greater RLS severity, the younger was onset age (35, 55). These findings will encourage further research to explore the characteristics of the high-risk population for RLS screening and assessment. In addition, the results of multiple clinical studies (refer to Supplementary Table 1) show that patients with high-risk PFO anatomical features, such as long tunnels, small angles, excessive atrial septal movement, and prominent eustachian tubes or Chiari networks, are also at relatively high risk of neurological disease, and these should be focused on.

## Pathogenesis of PFO-related neurological diseases

**Figure 1:**
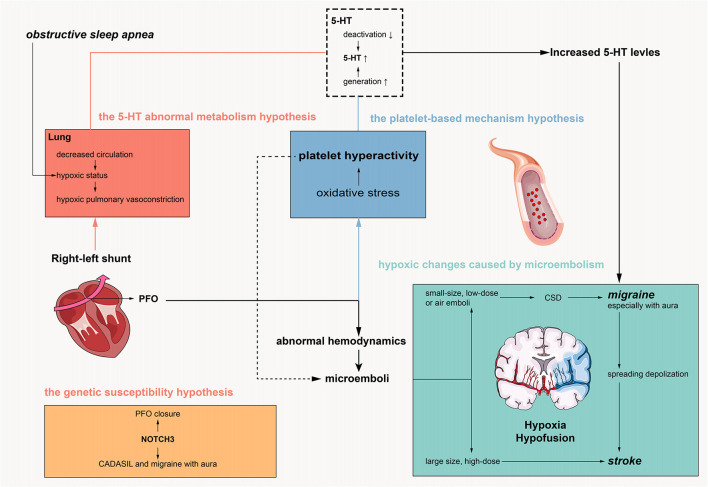
Pathogenesis of PFO related neurological disorders.

Although it was initially proposed that PFO is associated with these neurological diseases based on observational studies and clinical trials, mechanistic research remains at the hypothesis level. Figure 1 shows the research progress and hypothesized hypotheses on the pathogenesis of PFO-related neurological diseases. Currently, it is believed that the pathogenesis of PFO-related neurological diseases mainly includes ischemic hypoxic changes caused by microembolism, the 5-HT abnormal metabolism hypothesis, the platelet-based mechanism hypothesis, and the genetic susceptibility hypothesis.

The pathophysiology of PFO is characterized by abnormal right-to-left shunting of cardiac chambers, and abnormal hemodynamics in the left atrium and left atrial appendage easily lead to the formation of local microembolism (56). In addition, elevated platelet functions could be another reason for microembolism, which needs to be further investigated. It is currently believed that central system ischemia and hypoxia caused by PFO-related microembolism are the main mechanisms of nervous system diseases. Animal models have demonstrated that microembolism can cause pathological changes related to stroke and migraine when introduced into experimental animals (57, 58). Microembolization with larger sizes and higher doses can cause brain tissue injury, necrosis, neuroinflammation, and blood–brain barrier damage in mice (58). Microembolism of a smaller size and lower dose, especially air embolism, can cause cortical diffusion suppression (CSD) in mice, which is the hallmark pathological change of migraine with aura (57). It is worth mentioning that moderate microembolism can cause delayed stroke-like changes after inducing cortical diffusion inhibition, which may be related to the increased susceptibility to stroke caused by disseminated depolarization after migraine.

The hypothesis of abnormal metabolism of 5-HT is mainly related to the occurrence of migraine, which is characterized by increased production and decreased inactivation of 5-HT. Peripheral 5-HT production is mainly performed by platelets, and clinical studies have reported increased platelet activity in patients with PFO (59) and increased production of 5-HT (60). The inactivation process of peripheral 5-HT is mainly completed in the lung, while the atrial shunt of PFO is increased, leading to the decreased pulmonary circulation and decreased inactivation (60). However, the hypothesis of abnormal metabolism of 5-HT lacks sufficient evidence in animal experiments, and whether it is related to the occurrence of other neurological diseases is not clear.

The platelet-based mechanism hypothesis was reported in a recent article. Trabattoni et al. found that PFO had a prothrombotic potential sustained by an altered oxidative stress status in patients with migraine with aura, which could be associated with abnormal 5-HT levels (61). Furthermore, this association could be reverted after PFO closure by P2Y_12_-blockade. This hypothesis highlighted the role of oxidative stress in the pathology of PFO-related migraine. Future studies could provide a more detailed pathophysiologic pathway with basic experiments.

The genetic susceptibility hypothesis is still not mature. PFO closure is mediated by NOTCH signaling, particularly NOTCH3, a highly variable gene involved in CADASIL and migraine with aura (62). These genes could be related to the comorbidity of PFO and neurological diseases. However, the specific mechanism has not been confirmed by relevant tests.

In addition, whether there is a causal relationship between obstructive sleep apnea and PFO has not been determined. In terms of pathophysiology, they share a common mechanism, namely pulmonary circulation ischemia and hypoxia, which leads to a further increase in right-to-left shunting (63). Therefore, obstructive sleep apnea may aggravate PFO-related neurological disorders, which may be a new pathogenic mechanism of PFO, but no clinical studies have investigated this relationship.

With further research, proteomic-based studies have revealed more mechanistic hypotheses. The proteomic characteristics of serum albumin-binding proteins have recently been reported to change before and after PFO closure in patients with PFO-related stroke (64), which will be conducive to the exploration of targeted biomarkers. In the future, it may be more necessary to carry out multimodal in-depth studies combining peripheral blood and brain radiomics.

## Prevention and treatment of PFO-related neurological disorders

### Stroke

Secondary prevention is central to the management of patients with stroke. At present, specific preventive measures for PFO-related stroke are dominated by antiplatelet therapy (such as aspirin and clopidogrel), anticoagulation (mainly warfarin), and PFO closure. Several RCTs (The main information is shown in Table 2) provide clinical evidence for PFO-related stroke prevention.

**Table 2:** Summary of important information from randomized controlled clinical trials for the treatment of PFO-related stroke.

**Study**	**Study object**	**Interventions**	**Sample Size**	**Average age**	**Definition of large PFO**	**Moderate or Higher shunt (%)**	**Follow-up time (months)**	**Effectiveness**	**Safety**		
**Anticoagulation vs. antiplatelet therapy**											
PICSS study (28)	Cryptogenic stroke with PFO >18 years and < 85 years	Warfarin vs. Aspirin	203 (97/106)	57.9 ± 13.3	PFOs with ≥2 mm or with ≥10 microbubbles	41.4%	24	Year recurrent rates of ischemic stroke or death: 9.5 vs. 17.9%, *P* = 0.28	The rate of minor hemorrhage: 22.9 events/100 patient-years vs. 8.66 events/100 patient-years, *P* < 0.001		
Shariat et al. (65)	Undetermined causes of stroke with PFO >18 years	Warfarin vs. Aspirin	44(21/23)	60.6 ± 4.3/ 63.0 ± 4.7	Diameter ≥4 mm	31.7%	18	The rate of ischemic events or death: HR = 0.45; *P* = 0.259	The rate of Major bleeding: 4.3 vs. 9.5%, *P* = 0.501		
NAVIGATE ESUS trial (66)	Ischemic stroke with PFO >49 years at the time of the stroke	Rivaroxaban vs. Aspirin	7,213 (3,609/3,604)	66.9 ± 9.8/ 66.9 ± 9.8	NA	NA	11	The first recurrence rate of ischemic or hemorrhagic stroke or systemic embolism: 5.1 vs. 4.8%, *P* = 0.52	The rate of major bleeding: 1.8 vs. 0.7%, *P* < 0.001		
RE-SPECT ESUS2019 (67)	Undetermined source of stroke >18 years	Dabigatran vs. Aspirin	5,390 (2,695/2,695)	64.5 ± 11.4/ 63.9 ± 11.4	NA	NA	19	The recurrence rate of ischemic or hemorrhagic or unspecified type stroke: 6.6 vs. 7.7%, *P* = 0.10 Subgroup analysis: Patients with PFO consistent with the overall trial results	The rate of major bleeding: 1.7 vs 1.4% per year		
**Study**	**Study object**	**Sample Size**	**Average age**	**Device**	Definition of moderate or large RLS)^*^	**Moderate or** **Higher shunt (%)**	**Atrial septal Aneurysm (%)**	**Follow-up time** **(month)**	**Minimum duration of postoperative antiplatelet/ anticoagulant therapy (months)**	**Effectiveness**	**AF (%)**
**PFO closure plus antithrombotic therapy vs. antithrombotic medical Therapy**											
CLOSURE I 2012 (68)	Ischemic stroke or TIA with PFO between 18 and 60 years of age	909(447/462)	46.3 ± 9.6/ 45.7 ± 9.1	STARFlex 100%	≥10 bubbles (TEE)	55.9 vs 50.0	37.6 vs 35.7	24	24	Cumulative incidence of a composite of stroke or TIA or death: HR = 0.78, *P* = 0.37	5.7 vs. 0.7 (*P* < 0.001)
PC 2013 (69)	Ischemic stroke or TIA or peripheral thromboembolism with PFO < 60 years of age	414(204/210)	44.3 ± 10.2/ 44.6 ± 10.1	Amplatzer 100%	≥6 bubbles (TEE)	70.2 vs 60.9	23.0 vs 24.3	48.6	6	Cumulative incidence of a composite of death, nonfatal stroke, TIA, or peripheral embolism: HR = 0.63, *P* = 0.34	2.9 vs. 1.0 (*P* = 0.16)
RESPECT 2013–2017 (3)	Ischemic stroke or TIA with PFO between 18 and 60 years of age	980(499/481)	45.7 ± 9.7/ 46.2 ± 10.0	Amplatzer 100%	NA	NA	36.1 vs. 35.3	68.28	6	Cumulative incidence of a composite of stroke or death: HR = 0.55, *P* = 0.046	(0.48/100 patient-years) vs. (0.34/100 patient-years), *P* = 0.36
REDUCE 2017 (4)	Cryptogenic ischemic stroke with PFO 18 to 59 years of age	664(441/223)	45.4 ± 9.3/ 44.8 ± 9.6	GORE HELEX39% Cardioform61%	≥6 bubbles (TEE)	81.9 vs 80.1	20.4 vs NA	38	NA	•Rate of recurrence of stroke: HR = 0.23, *P* = 0.002 •The incidence of new brain infarctions:RR = 0.44, *P* = 0.02	6.6 vs. 0.4, *P* < 0.001
CLOSE 2017 (5)	Ischemic stroke with PFO 16 to 60 years of age	663(238/425)	42.9 ± 10.1/ 54 ± 12	NA	≥30 bubbles (large) (TEE)	90.8 vs NA	NA	63.6	3	Occurrence of fatal or non-fatal stroke: HR = 0.03, *P* < 0.001	4.6 vs. 0.9 (Antiplatelet-Only) *P =* 0.02
DEFENSE 2018 (70)	Ischemic stroke with high risk PFO	120(60/60)	49 ± 15/ 54 ± 12	Amplatzer 100%	NA	NA	8.3 vs 13.3	24	≥6	Cumulative incidence of a composite of stroke, vascular death, or Thrombolysis *P* = 0.013	3.33

* Shunt size was measured based on the number of microbubbles in the left atrium within three cycles of being seen in the right atrium on transthoracic or transesophageal echocardiography.

According to the guidelines, antiplatelet or anticoagulant therapy may be recommended in patients with low or uncertain associations between PFO and stroke and patients with contraindications for PFO closure (e.g., atrial fibrillation) (6, 15), but which drug is the best is still a matter of debate. No benefit of anticoagulant therapy over antiplatelet therapy has been found in the four RCTs conducted so far (Table 2). Even the latest ATTICUS trial found no significant difference in the efficacy of apixaban compared to aspirin for ESUS (reported from the last ESOC congress in 2022). Contrary to the results of multiple RCTs, two meta-analyses showed that the recurrence rate of stroke in the anticoagulant group was lower than that in the antiplatelet group (71, 72). It is possible that anticoagulant therapy can effectively prevent deep vein thrombosis, and the mechanism of action is more consistent with the hypothesis of the microemboli pathogenesis of PFO-related stroke. However, because of the risk of bleeding, there is insufficient evidence to support anticoagulants. It is still necessary to further combine the characteristics of the disease and the mechanism of action to seek the best drug therapy.

The efficacy of PFO closure in preventing stroke recurrence has basically reached consensus, and PFO closure has been strongly recommended for patients aged 18–60 years. However, there are still some disputes and problems to be solved urgently. (I) Definition of the best benefit group: In view of the inconsistent definition of the high-risk population for PFO in the existing RCT studies (73), at present, the determination of the optimal benefit population is still controversial. The SCAI guidelines still use a risk of paradoxical embolism (RoPE) score >7 as the recommended criterion (6). However, the French consensus holds that RoPE scoring has certain limitations (74). In the future, various diagnostic techniques should be used to explore more focused benefit groups by combining morphological characteristics of PFO, related anatomical characteristics of the adjacent atrial septum, and disease history. In addition, it is unclear whether patients older than 60 will benefit. (II) Duration of postoperative medication: Dual antiplatelet therapy is usually recommended within 1–6 months after PFO closure to prevent device thrombotic complications, but there is no accepted guideline for the duration of medication. A meta-regression analysis found that the duration of dual antiplatelet therapy after PFO closure was significantly associated with the incidence of TIA (15), while the effect on the recurrence of postoperative stroke was unknown. It is necessary to confirm whether the occasional postoperative bleeding was related to postoperative medication. (III) Device selection and postoperative atrial fibrillation (AF): New-onset atrial fibrillation is a common postoperative complication of PFO closure, mostly occurring in the first 45 days after surgery. Two recent studies reported a significantly higher incidence of postoperative AF (37% and 20.9%, respectively) than routinely reported (<6%) (75, 76), suggesting that the incidence of postoperative AF may be underestimated. Enough attention should be paid to exploring the risk factors of AF. Guedeney et al. found it was associated with older age, male sex, and device size. In addition, new-onset atrial fibrillation may be caused by device stimulation and endothelialization (77, 78). Therefore, the choice of device is also particularly important. At present, circumstantial evidence suggests that the use of Amplatzer (AMP) and GORE HELEX/CARDIOFORM Septal Occluder (GORE) is more effective and has a lower risk of postoperative atrial fibrillation than StarFlex (79). However, there is a lack of original research evidence for direct comparison between devices, and future real-world studies to explore the impact of devices may be considered. In addition, studies have shown that patients with lower RoPE scores have a higher risk of atrial fibrillation (80), which indicates that the cause of postoperative atrial fibrillation is not only related to devices but also that occult atrial fibrillation may be the mechanism of occurrence. At present, to reduce the risk of postoperative atrial fibrillation, the American Academy of Neurology guidelines strongly recommend electrocardiogram testing and atrial fibrillation evaluation for all patients considering PFO closure (6, 81).

### Migraine

As with PFO-related stroke, PFO-related migraine is treated primarily with medication and PFO closure. Medication therapy includes thienopyridine antiplatelet agents (e.g., clopidogrel and prasugrel) (82, 83) and nonthienopyridine P2Y_12_ inhibitors (e.g., ticagrelor). Although studies have reported that clopidogrel resistance is widespread (40% of migraine patients do not respond to treatment), Trabattoni et al. found the mechanisms of action of P2Y12 antagonists in the treatment of migraine with aura. Specifically, P2Y_12_ antagonists effectively inhibited the oxidative stress-induced platelet-associated tissue factor (TF) and reactive oxygen species (ROS) expression and on microvesicle information (61). This will bring new hope for the drug treatment of PFO-related migraine with aura.

For PFO closure in migraine, the results of three RCTs performed negatively in primary outcomes (shown in Table 3), but the PRIMA trial and PREMIUM trial obtained positive results in the migraine with aura subgroup analysis (9, 10). Based on this, PFO closure has been recommended for migraine with aura in the European position paper, but there are still issues that need further clarity, including (I) an appropriate crowd. Patients with refractory migraine combined with moderate-to-large RLS shunts in PFO were included in the three RCTs. The clinical symptoms of these patients were more severe, and it was difficult to improve the symptoms, which could be seen from the MIST study. To exclude two patients with long migraine days in the PFO closure group, the results of the study changed from negative to positive. In addition, the treatment effect for migraine without aura is not clear. (II) Selection of outcome indicators. The primary outcomes selected by the three RCTs were heterogeneous. Due to the lack of more clear or objective outcome indicators, migraine assessment is mainly based on a scale (such as the HIT-6 questionnaire), and the scores are largely affected by the variability of pain tolerance in different individuals (84). Thus, the results may be due to chance. Therefore, further consideration is needed to select more sensitive and convincing end points.

**Table 3:** Summary of important information from randomized controlled clinical trials for the treatment of PFO-related migraine.

**Study**	**Study object**	**Interventions**	**Device**	**Sample Size**	**Average age**	**Definition of moderate or** **higher RLS**	**Moderate or Higher shunt (%)**	**Atrial septal Aneurysm (%)**	**Follow-up time (months)**	**Effectiveness**	**Efficacy evaluation tool**	**Safety**
MIST 2008 (8)	• ≥5 headache days/ month • failed at least 2 lasses medication • 18–60 years of age	PFO closure vs. sham	STARFlex	147 (74/73)	44.3 ± 10.6/ 44.6 ± 10.4	≥10 bubbles (TTE)	37.7	34.0	6	Migraine headache cessation: (3 of 74) vs. (3 of 73), *P* = 0.51	(1) HIT-6 (2) SF-36v2 (3) MIDAS	pericardial effusion, retroperitoneal bleed
PRIMA 2016 (9)	• Minimum of 3 migraine attacks or 5–15 headache days/ month	PFO closure vs. Medical management	Amplatzer	107 (53/54)	44.1 ± 10.7/ 42.7 ± 11.0	NA	NA	NA	12	Reduction in monthly migraine days: –2.9 days vs. −1.7 days, *P* = 0.17	(1) MIDAS (2) SF-36v2 (3) Beck Depression Inventory (BDI)	AF, bleeding, retroperitoneal haematoma
PREMIUM 2017 (10)	• 6–14 headache days/ month • failed at least 3 lasses medication • severity RLS	PFO closure VS Medical management	Amplatzer	230 (123/107)	42.8 ± 10.3/ 43.7 ± 10.2	>100 bubbles (TCD)	100.0	NA	12	Responder rate: 38.5 vs. 32.0%	(1) MIDAS questionnaire (2) Beck Depression Inventory (BDI)	AF

### Other neurological diseases

Experience with PFO closure for other neurological diseases has been reported in observational studies (refer to Supplementary Table 1). The results of several clinical studies have demonstrated that PFO closure can improve sleep-disordered breathing and nocturnal oxygenation in patients with OSA. However, due to the small sample size, non-randomized controlled design, and low level of evidence, the effectiveness of PFO closure in improving clinical symptoms of OSA remains controversial (85, 86). In the field of decompression sickness research, a meta-analysis of four observational studies showed that PFO closure by divers can reduce the incidence of decompression sickness (87). However, the current international consensus primarily recommends behavioral and technical (B&T) changes to prevent DCS (88). If B&T changes are not possible or not effective, PFO closure can be proposed with shared decision-making underscoring the lack of evidence.

## Summary and future prospects

The prevalence of PFO in the general population is approximately 25%. Since the great shunt of RLS may be a high-risk factor for the occurrence and development of various PFO-related neurological diseases, it is necessary to conduct long-term follow-ups for such people to understand the law of the occurrence and development of diseases (such as exploring the peak age of various related neurological diseases). However, most of the current epidemiological studies are from patients with preexisting diseases, and few studies based on community populations may have a certain selection bias. There is still a lack of cluster random sampling-based representative samples to carry out prospective cohort studies. In addition, whether PFO has familial heritability is still unclear and needs to be further explored by pedigree studies. At the same time, studies have shown that the anatomical characteristics of PFO are not only a risk factor for the occurrence of diseases but also a key factor affecting the effect of PFO closure. Therefore, PFO-related studies are more dependent on accurate PFO diagnosis technology. It is urgent to develop more refined and standardized technical operation standards and norms to guide, and whether *in vitro* non-invasive testing techniques can be developed to improve patient compliance is still the focus of future research. For the correlation between PFO and various neurological diseases, since the mechanism hypothesis has not been confirmed and the therapeutic effect of PFO closure is still controversial, it is necessary to further explore the potential association based on basic and clinical research to scientifically guide the diagnosis and treatment of related diseases.

## Author contributions

FS, LS, HLi, and LC contributed to the conception and design of this study. YT, LH, HLiu, XL, LL, and WY searched and collected literature and materials. FS and LS wrote the first draft of the manuscript. LH, HLiu, and XL wrote sections of the manuscript. All authors contributed to the manuscript revision, read, and approved the submitted version.
