# Supplementary material for: Recent progress in patent foramen ovale and related neurological diseases: A narrative review
Source: Front Neurol. 2023 Mar 27;14:1129062. doi: 10.3389/fneur.2023.1129062 (PMC10084837; doi:10.3389/fneur.2023.1129062)
Supplement: Supplementary file 1 [file Table_1.docx]

Supplementary Material

**Recent progress in patent foramen ovale and related neurological diseases: A narrative review**

**Supplementary Table. Summary of clinical studies on PFO-related neurological diseases**

| **Clinical research on risk factors of PFO related stroke** | | | | | | | | | | | | | | | |
| --- | --- | --- | --- | --- | --- | --- | --- | --- | --- | --- | --- | --- | --- | --- | --- |
| **Study** | **Study type** | **Study object** | **Control group** | **Sample size （observation group /** **control group）** | **Average age （observation group /** **control group）** | **Main research results（observation group /** **control group）** | | | | | | **Conclusions** | | | |
| Holda MK, etal.2021(1) | prospective cohort | CS patients with PFO | Non-CS group with PFO | 199（106/93） | 45.6$\pm$11.8/48.1$\pm$14.5 | 1. PFO channel length change（OR=2.50，*P*<0.001） 2. PFO length/height ratio (OR=0.75.*P=*0.015) 3. Septum primum thickness (OR=0.34,*P*=0.013) 4. Septum secundum height(OR=0.91,*P*=0.013) 5. Atrial septal aneurys(OR=3.38,*P*=0.014)   （6）RLS large shunt(OR=2.49,*P*=0.022) | | | | | | The MorPFO score may help to identify high and moderate-risk PFO channels | | | |
| Bayar N, etal.2015(2) | retrospective cohort | CS and TIA patients with PFO | Asymptomatic patients with PFO | 156(64/92) | 43.8$\pm$8.6/37.8$\pm$9.3 | 1. Height of PFO（3.0 vs 2.0,*P*<0.001） 2. Thickness of septum secundum（5.0 vs 3.0,*P*<0.001） 3. Septal excursion distance（7.0 vs 4.0,*P*<0.001） 4. The ratio of length to height of PFO tunnel（3.0 vs 5.0,P<0.001） | | | | | | The long PFO, relatively large interatrial fluidity, thick septum pellucidum, and the presence of ASA may help to identify PFO patients aged ≤55 years | | | |
| Nakayama R, etal.2019(3) | retrospective cohort | CS patients planned for PFO closure | Migraine patients scheduled for PFO closure（Non- CS group） | 107（57/50） | 50$\pm$14/42$\pm$18 | （1）PFO tunnel（≥10mm vs <10mm(OR=3.16)）  （2）Hypermobile interatrial septum（OR=7.26）  （3）Eustachian valve or Chiari's network（OR=4.58）  （4）RLS shunt（large vs small，OR=3.87）  （5）PFO angle（≤10°vs ＞10°，OR=5.12） | | | | | | Long-tunnel PFO, the presence of hypermobile interatrial septum, the presence of prominent Eustachian valve or Chiari's network, the large right-to-left shunt during Valsalva maneuver, and low-angle PFO were independently related to CS | | | |
| Cerrato P,  etal.2002(4) | case-control | CS or TIA (including lacunar group and non-lacunar group) | Health control | 253（175/78） | 49.7/53.2 | 1. PFO frequency:31.4% vs 16.6%,P=0.02,N-LAC group vs LAC group=40.6% vs 17.4%,P=0.0005 ;   （2）Frequency of atrial septal aneurys :12% vs 1.3%,P=0.003 | | | | | | PFO and ASA are independent predictors of ischemic cerebrovascular disease, and PFO is independently associated with N-LAC stroke subtype | | | |
| Natanzon A, etal.2003(5) | case-control | CS patients with PFO | Non-CS group with PFO | 78（36/42） | 56.8$\pm$18.3/58.2$\pm$17.6 | （1）RLS shunt（*P*=0.004）  （2）The size of the overlap between septum primum and septum secundum（7.5 vs 9.9,*P*=0.026） | | | | | | Both anatomic and pathophysiologic mechanisms should be considered in determination of the potential clinical significance of a PFO | | | |
| Goel SS, etal.2009(6) | case-control | CS or TIA patients with PFO | Asymptomatic patients with PFO | 116（58/58） | 54$\pm$16/61$\pm$5 | （1）Proportion of large PFOs(>4 mm) :46% vs 17%，*P*<0.001  （2）Proportion of long tunnels (>1 cm):78% vs 55%，*P*<0.01  （3）Frequency of atrial septal aneurysm:45% vs 21%，*P*<0.005  （4）The frequencies of prominent Eustachian valves and Chiari’s network were not significantly different。 | | | | | | PFOs in patients with cryptogenic CVAs are larger, have longer tunnels, and are more frequently associated with atrial septal aneurysms | | | |
| Komar M,etal.2012(7) | case-control | CS patients with PFO | Asymptomatic patients with PFO | 176（88/88） | 36.1$\pm$16.2/35.7$\pm$14.2 | 1. PFO size:3.9 vs 1.3,*P*<0.0001 2. PFO tunnel length:14 vs 12，*P*<0.05 3. Frequency of atrial septal aneurysm:55% vs 15%,*P*<0.0001 4. Proportion of large PFOs:40% vs 2%,*P*<0.0001 | | | | | | PFO in symptomatic patients is larger in size, has a longer tunnel and is more frequently associated with atrial septal aneurysm | | | |
| **Clinical research on PFO closure in PFO related stroke** | | | | | | | | | | | | | | | |
| **Study** | **Study type** | **Study object** | **Interventions**  **（experimental group/control group）** | **Device** | **Sample Size**  **（experimental group/control group）** | **Average age （experimental group/control group）** | **Follow-up time（months）** | | **Minimum duration of postoperative antiplatelet/anticoagulant therapy (months)** | **Effectiveness** | | **Efficacy evaluation tool** | | **Residuals shunt** | **postoperative complications** |
| CLOSURE I 2012(8) | Multi-center RCT | (1)ischemic stroke or TIA with PFO  (2)between 18 and 60 years of age | PFO closure + antiplatelet drugs VS Drugs | STARFlex 100% | 909(447/462) | 46.3±9.6/  45.7±9.1 | 24 | | 24 | **Cumulative incidence of a composite of**  **stroke or TIA or death:**  HR=0.78，*P*=0.37 | | (1)MRI  (2) DWMRI | | NA | AF（5.7%）、bleeding |
| PC 2013(9) | Multi-center RCT | (1)ischemic stroke or TIA or peripheral thromboembolism with PFO  (2)< 60 years of age | PFO closure VS Drugs | Amplatzer 100% | 414（204/210) | 44.3±10.2/  44.6±10.1 | 48.6 | | 6 | **Cumulative incidence of a composite of**  **death, nonfatal stroke, TIA, or peripheral embolism**:  HR=0.63，*P*=0.34 | | (1)MRI or CT  (2)NIH Stroke Scale | | NA | AF（2.9%）、bleeding、myocardial infarction |
| RESPECT 2013-2017(10) | Multi-center RCT | (1)ischemic stroke or TIA with PFO  (2)between 18 and 60 years of age | PFO closure VS Drugs | Amplatzer 100% | 980(499/481) | 45.7±9.7/  46.2±10.0 | 68.28 | | 6 | **Cumulative incidence of a composite of**  **stroke or death:** HR=0.55，*P*=0.046 | | (1)MRI or CT  (2)NIH Stroke Scale  (3)Modified Rankin Scale and the Barthel Index | | NA | AF（0.48/100 patient-years） |
| REDUCE 2017(11) | Multi-center RCT | (1)cryptogenic ischemic stroke with PFO  (2)18 to 59 years of age | PFO closure + antiplatelet drugs VS Drugs | GORE HELEX39%、Cardioform61% | 664(441/223) | 45.4±9.3/  44.8±9.6 | 38 | | NA | **（1）rate of recurrence of stroke：**  HR=0.23,*P*=0.002  **（2）The incidence of new brain infarctions：**RR=0.44,*P*=0.02 | | MRI or CT | | NA | AF(6.6%)、bleeding |
| CLOSE 2017(12) | Multi-center RCT | (1)ischemic stroke with PFO  (2)16 to 60 years of age | PFO closure + antiplatelet drugs VS antiplatelet drugs VS anticoagulant drugs | NA | 663(238/425) | 42.9±10.1/  54±12 | 63.6 | | 3 | **occurrence of fatal or nonfatal stroke：**  HR=0.03，*P*<0.001 | | (1)MRI or CT  (2)modified Rankin Scale and the Barthel Index | | NA | AF(4.6%)、bleeding |
| DEFENSE 2018(13) | Multi-center RCT | ischemic stroke with high risk PFO | PFO closure VS Drugs | Amplatzer 100% | 120(60/60) | 49±15/  54±12 | 24 | | ≥6 | **Cumulative incidence of a composite of stroke,**  **vascular death, or Thrombolysis**  :*P*=0.013 | | MRI | | 1（1.67%） | AF（3.33%）、bleeding |
| Wahl et al, 2012(14) | prospective cohort | CS、TIA | PFO closure VS Drugs | NA | 308(150/158) | 50$\pm$12/50.7$\pm$13.3 | 180 | | NA | **Cumulative incidence of the composite of stroke, TIA, or peripheral embolism:**  HR=0.43，*P*=0.033 | | NA | | 4（3.9%） | Apparatus embolism、bleeding |
| Kim et al, 2018(15) | prospective cohort | CS、TIA | PFO closure VS Drugs | Amplatzer、GORE HELEX | 158(67/91) | 47.7$\pm$10.8/51.9$\pm$9..9 | 27.8 | | NA | Cumulative incidence of the composite of stroke, TIA：PFO closure VS Drugs=0.0% vs 6.6%,*P*=0.039 | | NA | | NA | NA |
| Alushi et al, 2014(16) | prospective cohort | CS、TIA | PFO closure VS Drugs | Amplatzer、Cardia Star | 418(262/156) | 48.5$\pm$13.8/52.3$\pm$13.1 | 70.8 | | 6 | Cumulative incidence of the composite of stroke, TIA：PFO closure VS Drugs=5.7% vs 5.1%,*P>*0.99 | | MRI or CT | | 5（1.9%） | AF、Blood clots in the device、endocarditis |
| Moon et al, 2016(17) | prospective cohort | CS | PFO closure VS Drugs | NA | 164(72/92) | 45.3$\pm$9.8/50.2$\pm$6.7 | 22 | | ≥3 | Cumulative incidence of the composite of stroke, TIA:=2.8% vs 2.2%,*P=*1.000） | | NA | | 8（14.5%） | NA |
| Harrer et al, 2006(18) | prospective cohort | CS | PFO closure VS Drugs | Amplatzer、CardioSEAL、PFOStar | 124(43/81) | 42.5$\pm$15.4/49.5$\pm$14.5 | 32 | | ≥6 | Annual stroke recurrence rates: PFO closure vs Drugs=2.9% vs 2.1%,*P*>0.05） | | MRI or CT | | 0（0.0%） | hematoma |
| Mazzucco et al, 2012(19) | prospective cohort | CS | PFO closure VS Drugs | Amplatzer、BioSTAR | 103(50/52) | 41.6$\pm$11.5/42.6$\pm$9.9 | 27.5 | | ≥6 | There was no difference in the risk of the composite of stroke, TIA | | MRI or CT | | 37% | bleeding |
| Pezzini et al, 2016(20) | prospective cohort | Stroke | PFO closure VS Drugs | Amplazer、CardioSEAL、STARFlex、GORE、BioSTAR、Premere、Figulla、ATRIASEPT | 521(206/315) | 35.3$\pm$7.4/35.7$\pm$6.8 | 36 | | NA | The composite recurrence rate of CS, TIA and peripheral embolism was not statistically significant. | | CT and/or MR angiography | | NA | AF |
| Casaubon et al, 2007(21) | prospective cohort | CS、TIA | PFO closure VS Antithrombotic drugs | CardioSEAL、Amplatzer | 121(60/61) | 46 | 32 | | Anticoagulation for 6 months and long-term antiplatelet | Recurrent rate of stroke: PFO closure group was lower than Antithrombotic drugs ，*P*=0.014） | | MRI or CT | | NA | AF、Thrombus 、hematoma |
| FORI Study, 2011(22) | prospective cohort | CS、TIA(age＜55 years old) | PFO closure VS Antithrombotic drugs | Amplatzer、STAR、Cardioseal/Starflex | 238(121/117) | 43.4$\pm$9.5/40.9$\pm$10.3 | 24 | | NA | Recurrent rate of stroke: PFO closure VS Antithrombotic drugs=0.8% vs 6.8%,*P*=0.018） | | NA | | 20（16.5%） | AF |
| Lee et al, 2010(23) | retrospective cohort | CS | PFO closure VS antiplatelet drugs VS anticoagulant drugs | Amplazer、CardioSEAL、 | 181(22/99/60) | 41$\pm$12/53$\pm$13 | 42 | | NA | unadjusted 5-year stroke recurrence-free survival rates were different | | NA | | NA | NA |
| Windecker et al, 2004(24) | retrospective cohort | CS | PFO closureVS Drugs | Amplazer、CardioSEAL、STARFlex | 308(150/158) | 50$\pm$12/51$\pm$13 | 27.6 | | NA | Recurrent rate of death, stroke, or TIA: PFO closure VS Drugs =8.5% vs 24.3%，*P*=0.05 | | NA | | 4(27%) | NA |
| Schuchlenz, 2005(25) | retrospective cohort | TIA or Stroke | PFO closure VS Aspirin VS coumadin | CardioSEAL、STARFlex、Amplatze | 280(167/66/47) | 44$\pm$11/46$\pm$13/50$\pm$12 | 31.2 | | NA | Recurrence rate of stroke or TIA: PFO closure VS Aspirin VS coumadin =0.6% vs 5.6% vs 13%,*P*<0.001 | | NA | | NA | retroperitoneal hematomas、device  embolizations、cardiac tamponade |
| Thanopoulos et al, 2006(26) | Case series | CS、TIA | PFO closure VS antiplatelet drugs | Amplatzer | 92(48/44) | 43$\pm$11/40$\pm$12 | 24 | | 6~9 | One-year incidence of TIA: PFO closure VS antiplatelet drugs =0% vs 6.8%,*P*=0.001 | | CT and/or MRI | | 4（9%） | NA |
| **Clinical research on drug treatment in PFO related stroke** | | | | | | | | | | | | | | | |
| **Study** | **Study type** | **Study object** | **Interventions**  **（experimental group/control group）** | **Sample Size （experimental group/control group）** | **Average age （experimental group/control group）** | **Follow-up time（months）** | | **Effectiveness** | | | **Efficacy evaluation tool** | | **Safety** | | |
| PICSS study2002(27) | RCT | (1)cryptogenic stroke with PFO  (2)>18 years and <85 years | Warfarin VS Aspirin | 203（97/106） | 57.9±13.3 | 24 | | 1. **year recurrent rates of ischemic stroke or death：** 2. 9.5% vs 17.9%,*P*=0.28 | | | CT or MRI | | 1. **year recurrent rates of ischemic stroke or death：** 2. 9.5% vs 17.9%,*P*=0.28 | | |
| Shariat,etal.  2013(28) | Single blind RCT | (1)undetermined causes of stroke with PFO  (2)>18 years | Warfarin VS Aspirin | 44(21/23) | 60.6±4.3/ 63.0±4.7 | 18 | | **The rate of ischemic events or death:**  HR= 0.45; *P* = 0.259 | | | NA | | **The rate of ischemic events or death:**  HR= 0.45; *P* = 0.259 | | |
| NAVIGATE ESUS trial(29) | RCT | (1)ischemic stroke with PFO  (2)>49 years at the time of the stroke | Rivaroxaban VS Aspirin | 7213(3609/3604) | 66.9±9.8/  66.9±9.8 | 11 | | **The first recurrence rate of ischemic or hemorrhagic stroke or systemic embolism：**5.1% vs 4.8%,*P*=0.52 | | | NA | | **The first recurrence rate of ischemic or hemorrhagic stroke or systemic embolism：**5.1% vs 4.8%,*P*=0.52 | | |
| RE-SPECT ESUS2019(30) | Multicenter, double-blind RCT | (1)undetermined source of stroke  (2)>18 years | Dabigatran VS Aspirin | 5390(2695/2695) | 64.5±11.4/ 63.9±11.4 | 19 | | **The recurrence rate of ischemic or hemorrhagic or unspecified type stroke:**  6.6% vs 7.7%,*P*=0.10  **Subgroup analysis: Patients with PFO consistent with the overall trial results** | | | NA | | **The recurrence rate of ischemic or hemorrhagic or unspecified type stroke:**  6.6% vs 7.7%,*P*=0.10  **Subgroup analysis: Patients with PFO consistent with the overall trial results** | | |
| CODICIA study2008(31) | prospective cohort | massive RLS of CS | Warfarin VS  Aspirin | NA | NA | NA | | The rate of recurrent strokes：Warfarin vs Aspirin = 6.6% vs 1.6%，*P*=0.18 | | | NA | | NA | | |
| K Nedeltchev2002(32) | retrospective cohort | TIA or Stroke | vitamin K antagonists  VS Aspirin/ clopidogrel | 159(79/80) | 50.7$\pm$13.5 | 29 | | Has no association between the treatment regimen and the risk for recurrence (p=0.99)，but there was a trend indicating an increased risk for stroke recurrence in Aspirin group | | | CT or MRI | | No bleeding complications were seen | | |
| **Clinical research on the correlation between PFO and** **migraine** | | | | | | | | | | | | | | | |
| **Study** | **Study type** | **Study object** | **Control group** | **Sample size （observation group /** **control group）** | **Average age （observation group /** **control group）** | **Outcome** | **Main research results** | | | | | **Conclusions** | | | |
| Ferrarini 2005(33) | retrospective cohort | migraine with aura | NA | 25 | 38.4 | The presence of PFO | 72% | | | | | the presence of arteriovenous (AV) shunts could represent a trigger for MA attacks as well as for stroke | | | |
| Rundek (NOMAS) 2008(34) | retrospective cohort | migraine | No Migraine | 1101 | 69 | The prevalence of PFO | no significantly difference between groups(14.6% vs 15.0%; P=0.9） | | | | | In this multiethnic, elderly, population-based cohort, PFO detected with transthoracic echocardiography and agitated saline was not associated with self-reported migraine | | | |
| Anzola 1999(35) | cross-sectional study | migraine patients | Non-migraine patients | MA+ 113  MA- 53  Non-migraine patients 25 | 33.7 | The prevalence of PFO | (1)MA（+） vs MA（-）OR=3.13,*P*=0.002;  (2)MA（+） vs control group：OR=3.66,*P*=0.01；  (3)MA（-） vs control group：OR=1.17 | | | | | Patency of the foramen ovale is associated with migraine with aura but not with migraine without aura | | | |
| Dalla Volta 2005(36) | cross-sectional study | migrainous patients | cluster headache(CH) | MA+ 260  MA- 74  CH 38 | 36.7 | The presence of PFO | MA(+) vs MA(-) vs CH=61.9% vs 16.2% vs 36.8% | | | | | There was a link between MA+, CH, and PFO, | | | |
| Domitrz 2007(37) | cross-sectional study | migraine patients | healthy control | MA+ 61  MA- 60  healthy control 65 | 36 | The presence of PFO | 1. MA(+) vs MA(-):*P*=0.0014; 2. MA(+) vs healthy control：*P=*0.0011; 3. MA(-) vs healthy control：*P*=0.087 | | | | | Possible association of migraine with aura and PFO | | | |
| Domitrz 2014(38) | cross-sectional study | migraine patients | healthy control | MA+ 62  MA- 96  healthy control 53 | 38$\pm$11 | The prevalence of PFO | (1)migraine patients vs healthy control：*P*=0.03; (2)MA vs MO：*P*=0.06; (3)MA vs healthy control:*P*=0.01. | | | | | Do not find any clear connection between PFO and migraine occurrence | | | |
| Guo 2014(39) | cross-sectional study | Chronic migraine（CM） | medication-overuse headache (MOH) | 261(159/102) | 38.25 | The prevalence of RLS | The prevalence of RLS :CM vs MOH=37% vs 31%,*P*=0.49 | | | | | PFO is thus unlikely to have a significant causal role in these chronic headaches | | | |
| Tang, Y. etal2022(40) | cross-sectional study | PFO group | No PFO group | 2640(880/1760) | 50.90 | The prevalence of migraine without aura | The prevalence of migraine without aura : PFO group VS No PFO group =12.83% vs 7.83%,*P*<0.0001 | | | | | a strong association between PFO and migraine  without aura, especially when the shunt is large | | | |
| Garg 2010(41) | case-control | migraine patients | healthy control | 288(144/144) | 41.5 | Presence of PFO | Presence of PFO： (1)migraine VS healthy control =26.4% vs 25.7%,OR=1.04，*P*=0.90；  (2)MA VS MO=26.8% vs 26.1%,OR=1.03，*P*=0.93 | | | | | Had no association between migraine headaches and the presence of PFO | | | |
| Kuper 2013(42) | case-control | migraine patients | healthy control | MA+ 42  MA- 44  healthy control 41 | NA | Prevalence of RLS | Differences did not reach statistical significance between the three groups (p = 0.564) | | | | | a trend towards higher prevalence of RLS with larger shunts in subjects with migraine with aura | | | |
| Schwerzmann 2005(43) | case-control | patients with migraine with aura | healthy control | 186(93/93) | NA | The presence of PFO and moderate-sized or large shunt | 1. The presence of PFO :MA+ VS healthy control=47% vs 17%，*P*<0.001;   (2) The presence of moderate-sized or large shunt :MA+ VS healthy control =38% vs 8%,*P*<0.001 | | | | | Shunt size is larger in migraineurs than controls | | | |
| Tatlidede 2007(44) | case-control | Migraine patients with intra-atrial right to left shunt | healthy control | 80(53/ 27) | NA | The percentages of PFO | MA+ VS MA- VS healthy control =66.7% vs 47.4% vs 22.2% | | | | | There were association between PFO and migraine, especially with aura | | | |
| **Clinical research on PFO closure in PFO related migraine** | | | | | | | | | | | | | | | |
| **Study** | **Study type** | **Study object** | **Interventions**  **（experimental group/control group）** | **Device** | **Sample Size**  **（experimental group/control group）** | **Average age （experimental group/control group）** | **Follow-up time（months）** | | **Maximum duration of postoperative antiplatelet/anticoagulant therapy (months)** | **Effectiveness** | | **Efficacy evaluation tool** | | **Residuals shunt** | **postoperative complications** |
| MIST 2008(45) | Multicenter, double-blind RCT | (1)≥5 headache days/month  (2)failed at least 2 lasses medication  (3)18 to 60 years of age | PFO closure VS sham | STARFlex | 147(74 /73) | 44.3±10.6/  44.6±10.4 | 6 | | NA | Migraine headache cessation:  （3 of 74）vs  （3 of 73），*P*=0.51 | | （1）HIT-6  （2） SF-36v2  （3）MIDAS questionnaire | | 4（5.4%） | pericardial effusion、retroperitoneal bleed |
| PRIMA 2016(46) | Multicenter, open-label RCT | minimum of 3 migraine attacks or 5~15 headache days/month | PFO closure VS Medical management | Amplatzer | 107(53/54) | 44.1±10.7/  42.7±11.0 | 12 | | NA | Reduction in monthly migraine days:  −2.9 days vs. −1.7 days , *P* = 0.17) | | （1）MIDAS questionnaire  （2）SF-36v2  （3）Beck Depression Inventory (BDI) | | NA | AF、bleeding、retroperitoneal haematoma |
| PREMIUM 2017(47) | Double-blind RCT | (1)6 to 14 headache days/month  (2)failed at least 3 lasses medication  (3)severity RLS | PFO closure VS Medical management | Amplatzer | 230(123/107) | 42.8±10.3/  43.7±10.2 | 12 | | NA | Responder rate,:  38.5% vs 32.0% | | （1）MIDAS questionnaire  （2）Beck Depression Inventory (BDI) | | 22(18%) | AF |
| Xing, 2016(48) | NRCT | substantial or severe migraineurs with a right-to-left shunt (RLS) (grade II–IV) | Transcatheter closure group VS control group | cardio - fix | 241(125/116) | 39.0± 12.9  38.3 ± 12.2 | 12 | | 6 | HIT-6 score(49.1 vs 57.5,*P*<0.001) | | HIT-6 | | 18(14.4%) | Pericardial tamponade |
| Jesurum,2008(49) | prospective cohort | patients with presumed paradoxical embolism and  Migraine Aura who underwent PFO closure | PFO closure | Amplatzer、Cardioseal、 | 77（55/22） | 47±12/46±10 | 18 | | 6 | Migraineurs with aura were 4.5 times more likely to experience migraine relief than  migraineurs without aura(*P*=0.02) | | NA | | 23(34%) | NA |
| Luermans,2008(50) | prospective cohort | Migraine patients underwent a percutaneous closure | Before-after control | Amplatzer、Cardioseal、Cardiastar、Helex | 92 | 51.6±12.3 | 6 | | 6 | prevalence of migraine in this group decreased from 28.6% to 10.7% (P = 0.001) | | Headache questionnaire | | NA | inguinal haematoma |
| Rigatelli  et al.2012(51) | prospective cohort | with severe, disabling, medication-refractory migraine and documented PFO underwent transcatheter PFO closure | Before-after control | Amplatzer、Premere、Biostar | 80 | 38.9±5.8 | 24~76 | | NA | (1)87.5% patients reported improved migraine symptomatology;(2)96.8% migraine with aura patients were definitively cured | | MIDAS | | NA | NA |
| Vigna 2009(52) | prospective cohort | moderate/severe migraine with PFO | PFO closure group vs Control group | Amplatzer/Cardia/  CardioSEAL/STARFlex | 82(53/29) | 42.0± 10  43 ± 11 | 16±7 | | 6 | Migraine disappeared in 34% of the closure group patients and 7% of  controls (p= 0.007) | | NA | | 3 (6%) | NA |
| Azarbal et al., 2005(53) | prospective cohort | Migraine patients | Migraine patients | CardioSEAL、Amplatzer | 89 | 49± 13 | 12 | | NA | MHA disappeared  completely in 75% of patients with MHA and aura and in 31% of patients with MHA  without aura. | | MIDAS | | 12(13.5%) | NA |
| Kimmelstiel 2007(54) | prospective cohort | Migraine patients | PFO closure VS Open  PFO VS No PFO | Amplatzer | PFO closure:41  Open PFO:63  No PFO:65 | 54  62  63 | NA | | NA | PFO closure reduced migraine MIDAS scores compared with the other two groups（*P*<0.001;*P*=0.035） | | MIDAS | | NA | NA |
| Dubiel,2008  (55) | retrospective cohort | patients with presumed paradoxical embolism underwent percutaneous transcatheter closure | Before-after control | Amplatzer、SEAL、STARFlex | 46 | 44±13.5 | 39.6±23.9 | | 6 | number of accompanying symptoms were significantly reduced (P<0.000) | | Self-made questionnaire | | 1（2.2%） | NA |
| Eyal.  et al.2020(56) | retrospective cohort | Migraine patients underwent transcatheter PFO closure | Before-after control | Amplatzer、CardioSEAL | 110 | 32.7±11.5 | 38.4 | | 6 | 87.0% of symptoms were significantly relieved and 48% were completely eliminated. | | Headache questionnaire | | 26% | NA |
| Biasco 2014(57) | retrospective cohort | Migraine with PFO | PFO closure VS medical treatment | Amplatzer/Cardia/  Others | 217(89/128) | 46.4 ± 12.7  47.1 ± 12.3 | 12 | | 6 | 1. MIDAS score decreased:*P*=0.204 2. number of patients reported a perceived   clinical benefit or the disappearance of migraine（*P*<0.001） | | MIDAS | | 16 (24%) | endocarditis |
| He, 2019(58) | retrospective cohort | migraineurs with RLS | PFO closure group VS non-PFO closure group | NA | 192(91/101) | 37.1± 12.8  39.2± 12. 1 | 60 | | NA | HIT-6 scores:  36 vs 52,*P*<0.001 | | HIT-6 | | NA | NA |
| Wahl  et al.2010(59) | retrospective cohort | patients undergoing percutaneous PFO closure | Before-after control | Amplatzer | 150 | 51.6±11 | 60 | | 6 | 1. mean headache frequency:(from 233/month to 13/month; p<0.001),; 2. The prevalence of any migraine headaches (from 100% to 66%; p<0.001) 3. the number of patients taking any migraine medication (from 90% to 50%; p<0.001) | | A structured questionnaire about Headache | | 14（9%） | NA |
| Rigatelli  et al.2010(60) | case-control | Migraine with PFO | PFO closure VS Medical therapy | Amplatzer、Premere | 86(40/46) | 38.9±5.8  40.0±3.7 | 29.2±14.8 | | NA | Mean MIDAS scores（8.3 vs 7.8,*P*<0.03） | | MIDAS | | NA | NA |
| **Clinical research on drug treatment in PFO related migraine** | | | | | | | | | | | | | | | |
| **Study** | **Study type** | **Study object** | **Interventions**  **（experimental group/control group）** | **Sample Size （experimental group/control group）** | **Average age （experimental group/control group）** | **Follow-up time（months）** | | **Effectiveness** | | | **Efficacy evaluation tool** | | **Safety** | | |
| Guo Y, et al.2020^(61)^ | Single arm trial | drug-refractory migraineurs with PFO | Clopidogrel（Before-after control） | 30 | NA | 3、6 | | （1）Headache Frequencies lower (*P*=0.003)（2）Headache attack durations lower(*P*=0.0049)  （3）VAS scores decreased(*P*<0.001)  （4）MIDASscores decreased(*P*=0.001) | | | VAS scores 、MIDAS scores | | **NA** | | |
| TRACTOR study.2018(62) | Single arm trial | MHA patients with PFO | Ticagrelor（single arm） | 40 | 36.3 ± 14.5 | 72 | | MHA responder rate：43% | | | NA | | Mild facial edema、Shortness of breath, transient | | |
| Sommer, R. J. etal.2018(63) | retrospective study | MHA/PFO patients | prasugrel、Clopidogrel（single arm） | 136 | 37.9 ± 14.6 | NA | | MHA responder rate：59% | | | NA | | mild bleeding、Cutaneous bruising | | |
| Spencer etal,2014(64) | retrospective study | Women with severe migraine and documented RLS | Clopidogrel（single arm） | 15 | 32.3 ± 11.9 | 11.9 | | MHA responder rate：87% | | | NA | | allergy | | |
| **Clinical research on correlation between PFO and other neurological diseases** | | | | | | | | | | | | | | | |
| **Study** | **Study type** | **Study object** | **Control group** | **Sample size （observation group /** **control group）** | **Average age observation group /** **control group）** | **Outcome** | **Main research results** | | | | | **Conclusions** | | | |
| [Manolo Beelke](https://pubmed.ncbi.nlm.nih.gov/?term=Beelke+M&cauthor_id=14592325).etal 2003 (65) | case-control | OSA patients | Healthy control group | 167（78/89） | 53.0±12.0  48.0±9.0 | the prevalence of PFO | Case group vs control group=27% vs 15%，*P*＜0.05 | | | | | The prevalence of PFO in OSAS was significantly higher than that in normal control group | | | |
| [HShanoudy](https://pubmed.ncbi.nlm.nih.gov/?term=Shanoudy+H&cauthor_id=9440574).  etal (1998)(66) | case-control | OSA patients | Healthy control group | 72（48/24） | NA | the prevalence of PFO | Case group vs control group=69% VS 17%，*P*＜0.0001 | | | | | The prevalence of PFO is increased in OSA patients | | | |
| Xiaonan Li,etal(67) | case-control | OSA patients with PFO | PFO without OSA group 、Healthy control group | 171(48/61/62) | 53.17±9.69  48.62±13.15  43.77±13.54 | sleep quality  polysomnography | 1. Compared with the control group   poor sleep quality (P<0.001) 、efficiency (P<0.010), a decrease in the proportion of REM sleep, and a decrease in N3 sleep (P<0.050) and prolonged N2 sleep (P < 0.010). Lower nighttime minimum SpO2 and higher oxygen saturation index (p < 0.50)   1. Compared with the simple PFO group,   sleep latency (p < 0.001) was prolonged; wake after sleep onset (p < 0.001) and arousal times (p = 0.031) were increased; and sleep micro-arousal index (p = 0.037), periodic leg movement index (p = 0.024), and apnea hypopnea index (p < 0.001) were higher in the PFO with OSA group | | | | | Patients with PFO and OSA have poor sleep quality with changes in sleep stage and high occurrence rate of sleep disorders. | | | |
| HONĚK J,  etal(68) | case-control | Divers with a history of DCS | Healthy control group | 489（36/453） | 36.4±7.7  35.5±9.1 | the frequency of PFO | Case group vs Control group=97.2% vs 35.5%,*P*＜0.001, PFO grade 3 was a major risk factor for unprovoked DCS | | | | | a high-grade PFO was a major risk factor for unprovoked DCS in recreational scuba divers | | | |
| **Clinical research on PFO closure in other neurological diseases** | | | | | | | | | | | | | | | |
| **Study** | **Study type** | **Study object** | **Interventions**  **（experimental group/control group）** | **Device** | **Sample size** | **Average age (experimental group/control group）** | **Follow-up time（months）** | | **Maximum duration of postoperative antiplatelet/anticoagulant therapy (months)** | **Effectiveness** | | | | **Efficacy evaluation tool** | **postoperative complications** |
| Rimoldi,S. F.2015(69) | NRCT | OSAS | PFO closure VS non-PFO | Amplatzer | 40 | 54±12/54±9 | 3 | | NA | （1）oxygen desaturation index:-7.6$\pm$ 16.6 vs 7.6$\pm$ 17.0 events/h, P=0.01  （2）apnea and hypopnea index:-7.9 $\pm$10.4 vs 4.7$\pm$ 13.1events/h， P=0.0009  （3）the propotion of patients with severe OSA decreased:79% vs 21%，P=0.007 | | | | Electroencephalograms、  electrooculograms 、electromyograms、  finger pulse oximetry | NA |
| PCOSAstudy.  2017(70) | prospective cohort | OSAS+ Moderate RLS shunts | Comparison before and after PFO closure | GORE | 26 | 51.6 | 6 | | 6 | 1. Epworth Sleepiness Scale score（13 vs 6,P<0.001） 2. Sleep Apnea Quality of Life Index(3.4 vs 4.4,P<0.001) 3. there were no statistically significant in oxygen desaturation index、Apnea Hypopnea Index and 6MWT | | | | (1) 6MWT  (2) ESS  (3)SF-36  (4) Sleep apnoea quality of life index  (5) Functional outcome of sleep questionnaire | NA |
| Shaikh ZF2013(71) | case series | OSAS+PFO | Comparison before and after PFO closure | NA | 6 | 54 | 12 | | NA | There was no significant difference between preoperative and preoperative changes in oxygen-desaturation index（P=0.92）、percentage of nocturnal arterial oxygen saturation < 90%(P=0.35) | | | | (1) 6MWT  (2) ESS  (3) HIT6  (4) SF-36v2 | Palpitations、hematochezia |
| Billinger  et al.2011(72) | prospective cohort | diver with a history of severe decompression | PFO closure VS PFO no closure VS no PFO | Amplatzer | 104 | NA | 63.6 | | NA | DCI events :PFO closure vs PFO not closure(P=0.045) | | | | MRI | NA |
| Anderson  et al.2019(73) | prospective cohort | Adult diver with PFO | PFO closure groupVS Conservative group | NA | 77 | 45.5/52 | 60~72 | | NA | The incidence of DCS decreased significantly after intervention, and the effect was more obvious in patients with larger PFO, while there was no change in conservative group | | | | NA | Bleeding, AF, palpitations, premature atrial and ventricular contractions |
| Honek  et al.2022(74) | prospective and retrospective cohort | Professional divers (including RLS Grade 1-3 and no PFO) | PFO closure VS PFO not closure | Amplatzer/ Occlutech | 829 | 35.4±10 | 78 | | NA | DCS incidence   1. PFO closure in low-grade group vs control group: HR= 3.965; *P* = 0.169; 2. PFO closure in the high-grade group vs control group :HR=26.170; *P* < 0.0001 | | | | NA | bleeding |
| Koopsen  et al.2018(75) | retrospective cohort | Decompression disease of unknown origin | PFO/ASD closed VS PFO/ASD not closed VS PFO/ASD absent | Amplatzer/  Occlutech | 62 | 38.3 | 81.6 | | NA | There was no recurrence of severe DCS in the PFO closure group | | | | NA | NA |

# References

1. Hołda MK, Krawczyk-Ożóg A, Koziej M, Kołodziejczyk J, Sorysz D, Szczepanek E, et al. Patent Foramen Ovale Channel Morphometric Characteristics Associated with Cryptogenic Stroke: The Morpfo Score. *Journal of the American Society of Echocardiography : official publication of the American Society of Echocardiography* (2021) 34(12):1285-93.e3. Epub 2021/08/15. doi: 10.1016/j.echo.2021.07.016.

2. Bayar N, Arslan Ş, Çağırcı G, Erkal Z, Üreyen Ç M, Çay S, et al. Assessment of Morphology of Patent Foramen Ovale with Transesophageal Echocardiography in Symptomatic and Asymptomatic Patients. *Journal of stroke and cerebrovascular diseases : the official journal of National Stroke Association* (2015) 24(6):1282-6. Epub 2015/04/25. doi: 10.1016/j.jstrokecerebrovasdis.2015.01.036.

3. Nakayama R, Takaya Y, Akagi T, Watanabe N, Ikeda M, Nakagawa K, et al. Identification of High-Risk Patent Foramen Ovale Associated with Cryptogenic Stroke: Development of a Scoring System. *Journal of the American Society of Echocardiography : official publication of the American Society of Echocardiography* (2019) 32(7):811-6. Epub 2019/05/28. doi: 10.1016/j.echo.2019.03.021.

4. Cerrato P, Imperiale D, Priano L, Mangiardi L, Morello M, Marson AM, et al. Transoesophageal Echocardiography in Patients without Arterial and Major Cardiac Sources of Embolism: Difference between Stroke Subtypes. *Cerebrovascular diseases (Basel, Switzerland)* (2002) 13(3):174-83. Epub 2002/03/27. doi: 10.1159/000047772.

5. Natanzon A, Goldman ME. Patent Foramen Ovale: Anatomy Versus Pathophysiology--Which Determines Stroke Risk? *Journal of the American Society of Echocardiography : official publication of the American Society of Echocardiography* (2003) 16(1):71-6. Epub 2003/01/07. doi: 10.1067/mje.2003.34.

6. Goel SS, Tuzcu EM, Shishehbor MH, de Oliveira EI, Borek PP, Krasuski RA, et al. Morphology of the Patent Foramen Ovale in Asymptomatic Versus Symptomatic (Stroke or Transient Ischemic Attack) Patients. *The American journal of cardiology* (2009) 103(1):124-9. Epub 2008/12/23. doi: 10.1016/j.amjcard.2008.08.036.

7. Komar M, Podolec P, Przewłocki T, Wilkołek P, Tomkiewicz-Pająk L, Motyl R. Transoesophageal Echocardiography Can Help Distinguish between Patients with "Symptomatic" and "Asymptomatic" Patent Foramen Ovale. *Kardiologia polska* (2012) 70(12):1258-63. Epub 2012/12/25.

8. Furlan AJ, Reisman M, Massaro J, Mauri L, Adams H, Albers GW, et al. Closure or Medical Therapy for Cryptogenic Stroke with Patent Foramen Ovale. *The New England journal of medicine* (2012) 366(11):991-9. Epub 2012/03/16. doi: 10.1056/NEJMoa1009639.

9. Meier B, Kalesan B, Mattle HP, Khattab AA, Hildick-Smith D, Dudek D, et al. Percutaneous Closure of Patent Foramen Ovale in Cryptogenic Embolism. *The New England journal of medicine* (2013) 368(12):1083-91. Epub 2013/03/22. doi: 10.1056/NEJMoa1211716.

10. Saver JL, Carroll JD, Thaler DE, Smalling RW, MacDonald LA, Marks DS, et al. Long-Term Outcomes of Patent Foramen Ovale Closure or Medical Therapy after Stroke. *The New England journal of medicine* (2017) 377(11):1022-32. Epub 2017/09/14. doi: 10.1056/NEJMoa1610057.

11. Søndergaard L, Kasner SE, Rhodes JF, Andersen G, Iversen HK, Nielsen-Kudsk JE, et al. Patent Foramen Ovale Closure or Antiplatelet Therapy for Cryptogenic Stroke. *The New England journal of medicine* (2017) 377(11):1033-42. Epub 2017/09/14. doi: 10.1056/NEJMoa1707404.

12. Mas JL, Derumeaux G, Guillon B, Massardier E, Hosseini H, Mechtouff L, et al. Patent Foramen Ovale Closure or Anticoagulation Vs. Antiplatelets after Stroke. *The New England journal of medicine* (2017) 377(11):1011-21. Epub 2017/09/14. doi: 10.1056/NEJMoa1705915.

13. Lee PH, Song JK, Kim JS, Heo R, Lee S, Kim DH, et al. Cryptogenic Stroke and High-Risk Patent Foramen Ovale: The Defense-Pfo Trial. *Journal of the American College of Cardiology* (2018) 71(20):2335-42. Epub 2018/03/17. doi: 10.1016/j.jacc.2018.02.046.

14. Wahl A, Jüni P, Mono ML, Kalesan B, Praz F, Geister L, et al. Long-Term Propensity Score-Matched Comparison of Percutaneous Closure of Patent Foramen Ovale with Medical Treatment after Paradoxical Embolism. *Circulation* (2012) 125(6):803-12. Epub 2012/01/13. doi: 10.1161/circulationaha.111.030494.

15. Kim M, Kim S, Moon J, Oh PC, Park YM, Shin DH, et al. Effect of Patent Foramen Ovale Closure for Prevention on Recurrent Stroke or Transient Ischemic Attack in Selected Patients with Cryptogenic Stroke. *Journal of interventional cardiology* (2018) 31(3):368-74. Epub 2017/08/24. doi: 10.1111/joic.12430.

16. Alushi B, Biasco L, Orzan F, Omedé P, Sciuto F, Moretti C, et al. Patent Foramen Ovale Treatment Strategy: An Italian Large Prospective Study. *Journal of cardiovascular medicine (Hagerstown, Md)* (2014) 15(10):761-8. Epub 2014/07/01. doi: 10.2459/jcm.0000000000000138.

17. Moon J, Kang WC, Kim S, Oh PC, Park YM, Chung WJ, et al. Comparison of Outcomes after Device Closure and Medication Alone in Patients with Patent Foramen Ovale and Cryptogenic Stroke in Korean Population. *Yonsei medical journal* (2016) 57(3):621-5. Epub 2016/03/22. doi: 10.3349/ymj.2016.57.3.621.

18. Harrer JU, Wessels T, Franke A, Lucas S, Berlit P, Klötzsch C. Stroke Recurrence and Its Prevention in Patients with Patent Foramen Ovale. *The Canadian journal of neurological sciences Le journal canadien des sciences neurologiques* (2006) 33(1):39-47. Epub 2006/04/06. doi: 10.1017/s0317167100004674.

19. Mazzucco S, Bovi P, Carletti M, Tomelleri G, Golia G, Stegagno C, et al. A Model of Multi-Disciplinary Approach to the Diagnosis and Treatment of Young Patients with Cryptogenic Stroke and Patent Foramen Ovale. *Cardiology in the young* (2012) 22(3):327-34. Epub 2012/02/22. doi: 10.1017/s1047951112000029.

20. Pezzini A, Grassi M, Lodigiani C, Patella R, Gandolfo C, Zini A, et al. Propensity Score-Based Analysis of Percutaneous Closure Versus Medical Therapy in Patients with Cryptogenic Stroke and Patent Foramen Ovale: The Ipsys Registry (Italian Project on Stroke in Young Adults). *Circulation Cardiovascular interventions* (2016) 9(9). Epub 2016/09/02. doi: 10.1161/circinterventions.115.003470.

21. Casaubon L, McLaughlin P, Webb G, Yeo E, Merker D, Jaigobin C. Recurrent Stroke/Tia in Cryptogenic Stroke Patients with Patent Foramen Ovale. *The Canadian journal of neurological sciences Le journal canadien des sciences neurologiques* (2007) 34(1):74-80. Epub 2007/03/14. doi: 10.1017/s0317167100005825.

22. Paciaroni M, Agnelli G, Bertolini A, Pezzini A, Padovani A, Caso V, et al. Risk of Recurrent Cerebrovascular Events in Patients with Cryptogenic Stroke or Transient Ischemic Attack and Patent Foramen Ovale: The Fori (Foramen Ovale Registro Italiano) Study. *Cerebrovascular diseases (Basel, Switzerland)* (2011) 31(2):109-16. Epub 2010/11/23. doi: 10.1159/000321334.

23. Lee JY, Song JK, Song JM, Kang DH, Yun SC, Kang DW, et al. Association between Anatomic Features of Atrial Septal Abnormalities Obtained by Omni-Plane Transesophageal Echocardiography and Stroke Recurrence in Cryptogenic Stroke Patients with Patent Foramen Ovale. *The American journal of cardiology* (2010) 106(1):129-34. Epub 2010/07/09. doi: 10.1016/j.amjcard.2010.02.025.

24. Windecker S, Wahl A, Nedeltchev K, Arnold M, Schwerzmann M, Seiler C, et al. Comparison of Medical Treatment with Percutaneous Closure of Patent Foramen Ovale in Patients with Cryptogenic Stroke. *Journal of the American College of Cardiology* (2004) 44(4):750-8. Epub 2004/08/18. doi: 10.1016/j.jacc.2004.05.044.

25. Schuchlenz HW, Weihs W, Berghold A, Lechner A, Schmidt R. Secondary Prevention after Cryptogenic Cerebrovascular Events in Patients with Patent Foramen Ovale. *International journal of cardiology* (2005) 101(1):77-82. Epub 2005/04/30. doi: 10.1016/j.ijcard.2004.03.005.

26. Thanopoulos BV, Dardas PD, Karanasios E, Mezilis N. Transcatheter Closure Versus Medical Therapy of Patent Foramen Ovale and Cryptogenic Stroke. *Catheterization and cardiovascular interventions : official journal of the Society for Cardiac Angiography & Interventions* (2006) 68(5):741-6. Epub 2006/10/14. doi: 10.1002/ccd.20868.

27. Homma S, Sacco RL, Di Tullio MR, Sciacca RR, Mohr JP. Effect of Medical Treatment in Stroke Patients with Patent Foramen Ovale: Patent Foramen Ovale in Cryptogenic Stroke Study. *Circulation* (2002) 105(22):2625-31. Epub 2002/06/05. doi: 10.1161/01.cir.0000017498.88393.44.

28. Shariat A, Yaghoubi E, Farazdaghi M, Aghasadeghi K, Borhani Haghighi A. Comparison of Medical Treatments in Cryptogenic Stroke Patients with Patent Foramen Ovale: A Randomized Clinical Trial. *Journal of research in medical sciences : the official journal of Isfahan University of Medical Sciences* (2013) 18(2):94-8. Epub 2013/08/06.

29. Hart RG, Sharma M, Mundl H, Kasner SE, Bangdiwala SI, Berkowitz SD, et al. Rivaroxaban for Stroke Prevention after Embolic Stroke of Undetermined Source. *The New England journal of medicine* (2018) 378(23):2191-201. Epub 2018/05/17. doi: 10.1056/NEJMoa1802686.

30. Diener HC, Sacco RL, Easton JD, Granger CB, Bernstein RA, Uchiyama S, et al. Dabigatran for Prevention of Stroke after Embolic Stroke of Undetermined Source. *The New England journal of medicine* (2019) 380(20):1906-17. Epub 2019/05/16. doi: 10.1056/NEJMoa1813959.

31. Serena J, Marti-Fàbregas J, Santamarina E, Rodríguez JJ, Perez-Ayuso MJ, Masjuan J, et al. Recurrent Stroke and Massive Right-to-Left Shunt: Results from the Prospective Spanish Multicenter (Codicia) Study. *Stroke* (2008) 39(12):3131-6. Epub 2008/09/27. doi: 10.1161/strokeaha.108.521427.

32. Nedeltchev K, Arnold M, Wahl A, Sturzenegger M, Vella EE, Windecker S, et al. Outcome of Patients with Cryptogenic Stroke and Patent Foramen Ovale. *Journal of neurology, neurosurgery, and psychiatry* (2002) 72(3):347-50. Epub 2002/02/28. doi: 10.1136/jnnp.72.3.347.

33. Ferrarini G, Malferrari G, Zucco R, Gaddi O, Norina M, Pini LA. High Prevalence of Patent Foramen Ovale in Migraine with Aura. *The journal of headache and pain* (2005) 6(2):71-6. Epub 2005/12/20. doi: 10.1007/s10194-005-0154-5.

34. Rundek T, Elkind MS, Di Tullio MR, Carrera E, Jin Z, Sacco RL, et al. Patent Foramen Ovale and Migraine: A Cross-Sectional Study from the Northern Manhattan Study (Nomas). *Circulation* (2008) 118(14):1419-24. Epub 2008/09/17. doi: 10.1161/circulationaha.108.771303.

35. Anzola GP, Magoni M, Guindani M, Rozzini L, Dalla Volta G. Potential Source of Cerebral Embolism in Migraine with Aura: A Transcranial Doppler Study. *Neurology* (1999) 52(8):1622-5. Epub 1999/05/20. doi: 10.1212/wnl.52.8.1622.

36. Dalla Volta G, Guindani M, Zavarise P, Griffini S, Pezzini A, Padovani A. Prevalence of Patent Foramen Ovale in a Large Series of Patients with Migraine with Aura, Migraine without Aura and Cluster Headache, and Relationship with Clinical Phenotype. *The journal of headache and pain* (2005) 6(4):328-30. Epub 2005/12/20. doi: 10.1007/s10194-005-0223-9.

37. Domitrz I, Mieszkowski J, Kamińska A. Relationship between Migraine and Patent Foramen Ovale: A Study of 121 Patients with Migraine. *Headache* (2007) 47(9):1311-8. Epub 2007/10/12. doi: 10.1111/j.1526-4610.2006.00724.x.

38. Domitrz I, Styczynski G, Wilczko J, Marczewska MM, Domitrz W, Kaminska A. An Association between Migraines and Heart Anomalies-True or False? A Heart Ultrasound Study Using Ctte in Migraine Patients and Control Participants. *Pain medicine (Malden, Mass)* (2014) 15(12):2156-60. Epub 2014/11/06. doi: 10.1111/pme.12607.

39. Guo S, Shalchian S, Gérard P, Küper M, Katsarava Z, Ashina M, et al. Prevalence of Right-to-Left Shunts on Transcranial Doppler in Chronic Migraine and Medication-Overuse Headache. *Cephalalgia : an international journal of headache* (2014) 34(1):37-41. Epub 2013/07/12. doi: 10.1177/0333102413497600.

40. Tang Y, Peng A, Peng B, He S, Zhao X, Zhu Y, et al. Association between Patent Foramen Ovale and Migraine without Aura: A Community-Based Cross-Sectional Study in China. *BMJ open* (2022) 12(3):e056937. Epub 2022/04/02. doi: 10.1136/bmjopen-2021-056937.

41. Garg P, Servoss SJ, Wu JC, Bajwa ZH, Selim MH, Dineen A, et al. Lack of Association between Migraine Headache and Patent Foramen Ovale: Results of a Case-Control Study. *Circulation* (2010) 121(12):1406-12. Epub 2010/03/17. doi: 10.1161/circulationaha.109.895110.

42. Küper M, Rabe K, Holle D, Savidou I, Dommes P, Frings M, et al. Prevalence of Cardiac Right Left Shunts in Migraine: A Population-Based Case-Control Study. *Neurological sciences : official journal of the Italian Neurological Society and of the Italian Society of Clinical Neurophysiology* (2013) 34(2):205-8. Epub 2012/03/01. doi: 10.1007/s10072-012-0986-0.

43. Schwerzmann M, Nedeltchev K, Lagger F, Mattle HP, Windecker S, Meier B, et al. Prevalence and Size of Directly Detected Patent Foramen Ovale in Migraine with Aura. *Neurology* (2005) 65(9):1415-8. doi: 10.1212/01.wnl.0000179800.73706.20.

44. Tatlidede AD, Oflazoğlu B, Celik SE, Anadol U, Forta H. Prevalence of Patent Foramen Ovale in Patients with Migraine. *Agri : Agri (Algoloji) Dernegi'nin Yayin organidir = The journal of the Turkish Society of Algology* (2007) 19(4):39-42. Epub 2007/12/27.

45. Dowson A, Mullen MJ, Peatfield R, Muir K, Khan AA, Wells C, et al. Migraine Intervention with Starflex Technology (Mist) Trial: A Prospective, Multicenter, Double-Blind, Sham-Controlled Trial to Evaluate the Effectiveness of Patent Foramen Ovale Closure with Starflex Septal Repair Implant to Resolve Refractory Migraine Headache. *Circulation* (2008) 117(11):1397-404. Epub 2008/03/05. doi: 10.1161/circulationaha.107.727271.

46. Mattle HP, Evers S, Hildick-Smith D, Becker WJ, Baumgartner H, Chataway J, et al. Percutaneous Closure of Patent Foramen Ovale in Migraine with Aura, a Randomized Controlled Trial. *European heart journal* (2016) 37(26):2029-36. Epub 2016/02/26. doi: 10.1093/eurheartj/ehw027.

47. Tobis JM, Charles A, Silberstein SD, Sorensen S, Maini B, Horwitz PA, et al. Percutaneous Closure of Patent Foramen Ovale in Patients with Migraine: The Premium Trial. *J Am Coll Cardiol* (2017) 70(22):2766-74. Epub 2017/12/02. doi: 10.1016/j.jacc.2017.09.1105.

48. Xing YQ, Guo YZ, Gao YS, Guo ZN, Niu PP, Yang Y. Effectiveness and Safety of Transcatheter Patent Foramen Ovale Closure for Migraine (Eastform) Trial. *Scientific reports* (2016) 6:39081. Epub 2016/12/15. doi: 10.1038/srep39081.

49. Jesurum JT, Fuller CJ, Kim CJ, Krabill KA, Spencer MP, Olsen JV, et al. Frequency of Migraine Headache Relief Following Patent Foramen Ovale "Closure" Despite Residual Right-to-Left Shunt. *The American journal of cardiology* (2008) 102(7):916-20. Epub 2008/09/23. doi: 10.1016/j.amjcard.2008.05.035.

50. Luermans JG, Post MC, Temmerman F, Thijs V, Schonewille WJ, Plokker HW, et al. Closure of a Patent Foramen Ovale Is Associated with a Decrease in Prevalence of Migraine: A Prospective Observational Study. *Acta cardiologica* (2008) 63(5):571-7. Epub 2008/11/19. doi: 10.2143/ac.63.5.2033223.

51. Rigatelli G, Dell'avvocata F, Cardaioli P, Giordan M, Braggion G, Aggio S, et al. Improving Migraine by Means of Primary Transcatheter Patent Foramen Ovale Closure: Long-Term Follow-Up. *American journal of cardiovascular disease* (2012) 2(2):89-95. Epub 2012/06/22.

52. Vigna C, Marchese N, Inchingolo V, Giannatempo GM, Pacilli MA, Di Viesti P, et al. Improvement of Migraine after Patent Foramen Ovale Percutaneous Closure in Patients with Subclinical Brain Lesions: A Case-Control Study. *JACC Cardiovascular interventions* (2009) 2(2):107-13. Epub 2009/05/26. doi: 10.1016/j.jcin.2008.10.011.

53. Azarbal B, Tobis J, Suh W, Chan V, Dao C, Gaster R. Association of Interatrial Shunts and Migraine Headaches: Impact of Transcatheter Closure. *Journal of the American College of Cardiology* (2005) 45(4):489-92. Epub 2005/02/15. doi: 10.1016/j.jacc.2004.09.075.

54. Kimmelstiel C, Gange C, Thaler D. Is Patent Foramen Ovale Closure Effective in Reducing Migraine Symptoms? A Controlled Study. *Catheterization and cardiovascular interventions : official journal of the Society for Cardiac Angiography & Interventions* (2007) 69(5):740-6. Epub 2007/02/14. doi: 10.1002/ccd.21025.

55. Dubiel M, Bruch L, Schmehl I, Liebner M, Winkelmann A, Stretz A, et al. Migraine Headache Relief after Percutaneous Transcatheter Closure of Interatrial Communications. *Journal of interventional cardiology* (2008) 21(1):32-7. Epub 2007/12/21. doi: 10.1111/j.1540-8183.2007.00316.x.

56. Ben-Assa E, Rengifo-Moreno P, Al-Bawardy R, Kolte D, Cigarroa R, Cruz-Gonzalez I, et al. Effect of Residual Interatrial Shunt on Migraine Burden after Transcatheter Closure of Patent foramen ovale. *JACC Cardiovascular interventions* (2020) 13(3):293-302. Epub 2020/02/08. doi: 10.1016/j.jcin.2019.09.042.

57. Biasco L, Infantino V, Orzan F, Vicentini S, Rovera C, Longo G, et al. Impact of Transcatheter Closure of Patent Foramen Ovale in the Evolution of Migraine and Role of Residual Shunt. *Journal of cardiology* (2014) 64(5):390-4. Epub 2014/04/10. doi: 10.1016/j.jjcc.2014.02.023.

58. He YD, Yan XL, Qin C, Zhang P, Guo ZN, Yang Y. Transcatheter Patent Foramen Ovale Closure Is Effective in Alleviating Migraine in a 5-Year Follow-Up. *Frontiers in neurology* (2019) 10:1224. Epub 2019/12/06. doi: 10.3389/fneur.2019.01224.

59. Wahl A, Praz F, Tai T, Findling O, Walpoth N, Nedeltchev K, et al. Improvement of Migraine Headaches after Percutaneous Closure of Patent Foramen Ovale for Secondary Prevention of Paradoxical Embolism. *Heart (British Cardiac Society)* (2010) 96(12):967-73. Epub 2010/06/12. doi: 10.1136/hrt.2009.181156.

60. Rigatelli G, Dell'Avvocata F, Ronco F, Cardaioli P, Giordan M, Braggion G, et al. Primary Transcatheter Patent Foramen Ovale Closure Is Effective in Improving Migraine in Patients with High-Risk Anatomic and Functional Characteristics for Paradoxical Embolism. *JACC Cardiovascular interventions* (2010) 3(3):282-7. Epub 2010/03/20. doi: 10.1016/j.jcin.2009.11.019.

61. Guo Y, Shi Y, Zhu D, Liu R, Qi Y, Luo G. Clopidogrel Can Be an Effective Complementary Prophylactic for Drug-Refractory Migraine with Patent Foramen Ovale. *Journal of investigative medicine : the official publication of the American Federation for Clinical Research* (2020) 68(7):1250-5. Epub 2020/08/28. doi: 10.1136/jim-2020-001342.

62. Reisman AM, Robbins BT, Chou DE, Yugrakh MS, Gross GJ, Privitera L, et al. Ticagrelor for Refractory Migraine/Patent Foramen Ovale (Tractor): An Open-Label Pilot Study. *Neurology* (2018) 91(22):1010-7. Epub 2018/11/28. doi: 10.1212/wnl.0000000000006573.

63. Sommer RJ, Nazif T, Privitera L, Robbins BT. Retrospective Review of Thienopyridine Therapy in Migraineurs with Patent Foramen Ovale. *Neurology* (2018) 91(22):1002-9. Epub 2018/11/28. doi: 10.1212/wnl.0000000000006572.

64. Spencer BT, Qureshi Y, Sommer RJ. A Retrospective Review of Clopidogrel as Primary Therapy for Migraineurs with Right to Left Shunt Lesions. *Cephalalgia : an international journal of headache* (2014) 34(11):933-7. Epub 2014/04/29. doi: 10.1177/0333102414523845.

65. Beelke M, Angeli S, Del Sette M, Gandolfo C, Cabano ME, Canovaro P, et al. Prevalence of Patent Foramen Ovale in Subjects with Obstructive Sleep Apnea: A Transcranial Doppler Ultrasound Study. *Sleep medicine* (2003) 4(3):219-23. Epub 2003/11/01. doi: 10.1016/s1389-9457(02)00256-3.

66. Shanoudy H, Soliman A, Raggi P, Liu JW, Russell DC, Jarmukli NF. Prevalence of Patent Foramen Ovale and Its Contribution to Hypoxemia in Patients with Obstructive Sleep Apnea. *Chest* (1998) 113(1):91-6. Epub 1998/01/24. doi: 10.1378/chest.113.1.91.

67. Li X, Liu C, Wu J, Jia Y, Li H, Yu H, et al. Analysis of the Characteristics of Sleep in Patients with Patent Foramen Ovale Complicated with Obstructive Sleep Apnea. *Sleep & breathing = Schlaf & Atmung* (2021) 25(4):1831-6. Epub 2021/01/14. doi: 10.1007/s11325-021-02289-0.

68. Honěk J, Šrámek M, Šefc L, Januška J, Fiedler J, Horváth M, et al. High-Grade Patent Foramen Ovale Is a Risk Factor of Unprovoked Decompression Sickness in Recreational Divers. *Journal of cardiology* (2019) 74(6):519-23. Epub 2019/07/01. doi: 10.1016/j.jjcc.2019.04.014.

69. Rimoldi SF, Ott S, Rexhaj E, de Marchi SF, Allemann Y, Gugger M, et al. Patent Foramen Ovale Closure in Obstructive Sleep Apnea Improves Blood Pressure and Cardiovascular Function. *Hypertension (Dallas, Tex : 1979)* (2015) 66(5):1050-7. Epub 2015/09/30. doi: 10.1161/hypertensionaha.115.06303.

70. Hoole SP, Hernández-Sánchez J, Davies WR, McNab DC, Calvert PA, Rana BS, et al. Effects of Patent Foramen Ovale Closure on Obstructive Sleep Apnea Syndrome: Pcosa Study. *The Canadian journal of cardiology* (2017) 33(12):1708-15. Epub 2017/11/28. doi: 10.1016/j.cjca.2017.09.005.

71. Shaikh ZF, Jaye J, Ward N, Malhotra A, de Villa M, Polkey MI, et al. Patent Foramen Ovale in Severe Obstructive Sleep Apnea: Clinical Features and Effects of Closure. *Chest* (2013) 143(1):56-63. Epub 2012/08/28. doi: 10.1378/chest.12-0334.

72. Billinger M, Zbinden R, Mordasini R, Windecker S, Schwerzmann M, Meier B, et al. Patent Foramen Ovale Closure in Recreational Divers: Effect on Decompression Illness and Ischaemic Brain Lesions During Long-Term Follow-Up. *Heart (British Cardiac Society)* (2011) 97(23):1932-7. Epub 2011/09/16. doi: 10.1136/heartjnl-2011-300436.

73. Anderson G, Ebersole D, Covington D, Denoble PJ. The Effectiveness of Risk Mitigation Interventions in Divers with Persistent (Patent) Foramen Ovale. *Diving and hyperbaric medicine* (2019) 49(2):80-7. Epub 2019/06/10. doi: 10.28920/dhm49.2.80-87.

74. Honěk J, Šrámek M, Honěk T, Tomek A, Šefc L, Januška J, et al. Screening and Risk Stratification Strategy Reduced Decompression Sickness Occurrence in Divers with Patent Foramen Ovale. *JACC Cardiovascular imaging* (2022) 15(2):181-9. Epub 2021/08/23. doi: 10.1016/j.jcmg.2021.06.019.

75. Koopsen R, Stella PR, Thijs KM, Rienks R. Persistent Foramen Ovale Closure in Divers with A history of Decompression Sickness. *Netherlands heart journal : monthly journal of the Netherlands Society of Cardiology and the Netherlands Heart Foundation* (2018) 26(11):535-9. Epub 2018/09/05. doi: 10.1007/s12471-018-1153-x.
